# Marfan's syndrome: an overview

**DOI:** 10.1590/S1516-31802010000600009

**Published:** 2010-12-02

**Authors:** Shi-Min Yuan, Hua Jing

**Affiliations:** I Postdoctoral Researcher, Department of Cardiothoracic Surgery, Jinling Hospital, School of Clinical Medicine, Nanjing University, Nanjing 210002, Jiangsu Province, People's Republic of China.; II Professor and Head, Department of Cardiothoracic Surgery, Jinling Hospital, School of Clinical Medicine, Nanjing University, Nanjing 210002, Jiangsu Province, People's Republic of China.

**Keywords:** Aortic aneurysm, Arachnodactyly, Connective tissue diseases, Marfan syndrome, Mitral valve prolapse, Aneurisma aórtico, Aracnodactilia, Doenças do tecido conjuntivo, Síndrome de Marfan, Prolapso da valva mitral

## Abstract

Marfan's syndrome is an autosomal dominant condition with an estimated prevalence of one in 10,000 to 20,000 individuals. This rare hereditary connective tissue disorder affects many parts of the body. The diagnosis of Marfan's syndrome is established in accordance with a review of the diagnostic criteria, known as the Ghent nosology, through a comprehensive assessment largely based on a combination of major and minor clinical manifestations in various organ systems and the family history. Aortic root dilation and mitral valve prolapse are the main presentations among the cardiovascular malformations of Marfan's syndrome. The pathogenesis of Marfan's syndrome has not been fully elucidated. However, fibrillin-1 gene mutations are believed to exert a dominant negative effect. Therefore, Marfan's syndrome is termed a fibrillinopathy, along with other connective tissue disorders with subtle differences in clinical manifestations. The treatment may include prophylactic β-blockers and angiotensin II-receptor blockers in order to slow down the dilation of the ascending aorta, and prophylactic aortic surgery. Importantly, β-blocker therapy may reduce TGF-β activation, which has been recognized as a contributory factor in Marfan's syndrome. The present article aims to provide an overview of this rare hereditary disorder.

## RESUMO

Síndrome de Marfan é uma condição autossômica dominante com prevalência estimada de 1 em 10.000 a 20.000 indivíduos. É uma rara desordem hereditária do tecido conjuntivo que afeta muitas partes do corpo. O diagnóstico da síndrome de Marfan é feito de acordo com uma revisão dos critérios diagnósticos conhecida como a nosologia Ghent, por meio de uma avaliação abrangente, em grande parte baseada em uma combinação de pequenas e grandes manifestações clínicas em vários sistemas de órgãos e na história familiar. Dilatação da raiz aórtica e prolapso da valva mitral são as principais apresentações entre as malformações cardiovasculares da síndrome de Marfan. A patogênese da síndrome de Marfan não foi totalmente esclarecida, mas acredita-se que mutações genéticas de fibrillina-1 exercem um efeito negativo dominante. Portanto, a síndrome de Marfan é denominada como fibrilinopatia, juntamente com outras desordens do tecido conectivo, com sutis diferenças nas manifestações clínicas. O tratamento pode incluir β-bloqueadores profiláticos e bloqueadores dos receptores da angiotensina II, a fim de retardar a dilatação da aorta ascendente e cirurgia profilática da aorta. De importância, a terapia com β-bloqueadores pode reduzir a ativação de TGF-β, que foi reconhecido como um fator contribuinte da síndrome de Marfan. O presente artigo visa proporcionar uma visão global desta rara desordem de hereditariedade.

## INTRODUCTION

Marfan's syndrome is an autosomal dominant condition with an estimated prevalence of one in 10,000 to 20,000 individuals. It is a rare hereditary connective tissue disorder that affects many parts of the body.^1^ There is no geographic, ethnic or gender predilection. It is also known as arachnodactyly, since this is one of the signs of Marfan's syndrome, which is characterized by abnormally long, slender or spidery fingers and toes.^2^ Liu Bei (A.D. 161-223), the founder of the Shu Han dynasty of the Three Kingdoms, and also the protagonist of “The Romance of Three Kingdoms”, one of the famous ancient Chinese works,^3^ and former American President Abraham Lincoln (1809-1865) are thought to have had Marfan's syndrome, in that they manifested several key clinical features.^3,4^ In 1896, Marfan's syndrome was first described in a 5.5 year-old girl by a French pediatrician named Antonin Marfan.^5^

A family history of Marfan's syndrome has been found to be present in 49% of the families of individuals with this condition.^6^ In about 25-30% of the patients, the disorder occurs without a positive family history, and gene mutation is likely to be taken into consideration.^7^ Most such severe cases appear to be due to sporadic mutation in a single germ cell of one parent. Many familial cases may have milder manifestations (for instance, mitral valve regurgitation is less frequent^8^) and a better prognosis, but may be more difficult to detect during infancy.^9^ The aortic root and arch diameters have been found to be significantly greater in patients with a family history than in those without such histories, and life expectancy has been found to be shorter.^10^ The current guideline for prophylactic aortic root replacement for patients with a positive family history is that the difference in aortic root diameter needs to be greater than 5 mm.^11^

## METHODS

A literature search was conducted in the Lilacs, PubMed, Embase and Cochrane Library databases using the search term “Marfan's syndrome”. A manual search of abstracts of articles was made to identify those relating to the topic. The results are presented in Table 1.

**Table 1.** Results from literature search in the major medical databases

Database	Search strategies		Results	
PubMed	Marfan's syndrome	5293 found	5293 related	1800 case reports 44 clinical trials 234 comparative studies 3 meta-analyses 6 randomized controlled trials 633 reviews 2573 others
Embase (Excerpta Medica databases) (1966-2010)	Marfan's syndrome	3801 found	3801 related	2371 articles 11 articles in press 0 Cochrane reviews 31 conference abstracts 147 conference papers 33 controlled clinical trials 111 editorials 207 letters 5 meta-analyses 69 notes 10 randomized controlled trials 654 reviews 105 short surveys 8 systematic reviews
Lilacs (Literatura Latino-Americana e do Caribe em Ciências da Saúde)	Marfan syndrome	116 found	116 related	6 case cohorts 53 case reports 1 classical article 1 clinical trial 1 comparative review study 7 prospective cohort studies 27 reviews 19 retrospective cohort studies 1 textbook
Cochrane	Marfan syndrome	8 found	8 related	8 clinical trials

## CLINICAL MANIFESTATIONS

The clinical manifestations of Marfan's syndrome become more evident with age. The most common symptom of Marfan's syndrome is myopia, and 60% of the individuals with Marfan's syndrome have ectopia lentis. Individuals who have Marfan's syndrome are also at higher risk of retinal detachment, glaucoma and early cataract formation. Other common symptoms of Marfan's syndrome involve the skeleton and connective tissue systems, including joint laxity, dolichostenomelia, pectus excavatum or pectus carinatum, and scoliosis.^12^ Cardiovascular malformations are the most life-threatening presentations of Marfan's syndrome. Aortic root dilation and mitral valve prolapse are significant clinical findings in patients with Marfan's syndrome,^9^ and have been recognized as being as prevalent as ocular defects in Marfan's syndrome.^13^ Sisk et al.^14^ found aortic root dilation and mitral valve prolapse in all their 13 cases of Marfan's syndrome, which presented at ages of less than four years. Other cardiovascular manifestations in infants may include coarctation of the aorta, atrial septal defect, patent ductus arteriosus, pulmonary artery stenosis, persistent left superior vena cava etc.^15^ The aorta is the principal site of the lesions; particularly, the aortic root tends to develop dilation, aneurysm and dissection. In addition, mitral valve prolapse may also be observed.^16^ Aortic root dilation was found in 60% of a series of patients with Marfan's syndrome (74% males, 33% females) while mitral valve prolapse was found in 91% (87% males, 100% females).^13^ The histopathology of the aortic wall is characterized by widespread fragmentation of the elastin component, and the elastin fibers are often thin. Aortic dissection, which is common in Marfan's syndrome, is usually due to an intimal tear in the proximal ascending aorta, with dissection involving the sinotubular junction and aortic sinuses, thus resulting in prolapse of one or more commissures.^16^ The principal pathological findings from the mitral valve are annular dilation, fibromyxomatous changes to the leaflets and chordae, elongation and rupture of chordae and deposition of calcium.^17^

## PATHOGENESIS

The pathogenesis of Marfan's syndrome has not been fully elucidated. However, fibrillin-1 gene mutations are believed to exert a dominant negative effect.^18^ Fibrillin is an extracellular matrix protein that forms a major component of microfibrils of the extracellular matrix of both elastic and non-elastic connective tissues and which is essential for normal elastic fibrillogenesis.^19^ The fibrillin-1 gene contains 65 exons, located at chromosome 15q-21.1. Fibrillin-1 mutation disrupts microfibril formation, thereby resulting in fibrillin protein abnormalities, and subsequently weakening the connective tissue.^20^

Changes to the transforming growth factor-beta (TGF-β)-signaling pathway may lead to diverse Marfan phenotypes.^21^ The gene defect ultimately causes erratic binding between the fibrillin and connective tissue matrix. Mutations to transforming growth factor-beta receptor 2 (TGFβR2) in patients with Marfan's syndrome type II (MFS2 mapped at 3p24.2-p25) have demonstrated alternative evidence for abnormal TGF-β signaling in the pathogenesis of Marfan's syndrome.^22^

Gene mutation may be absent in Marfan's syndrome, and patients with the gene mutation do not necessarily develop the clinical manifestations of Marfan's syndrome. The current theories support the notion that fibrillin mutations have an impact on the development of Marfan's syndrome. However, only 28%-66% of the patients with Marfan's syndrome have been found to have fibrillin-1 mutations.^23,24^ Moreover, fibrillin-1 mutations have also been found in patients with familial aortic aneurysms, mitral valve prolapse or Marfan-like skeletal abnormalities, such as scoliosis or pectus excavatum.^18^ They have also been found in a wide range of connective tissue disorders collectively known as fibrillinopathies, ranging from mild phenotypes, such as isolated ectopia lentis, to severe disorders including neonatal Marfan's syndrome, which generally leads to death within the first two years of life.^25^

## DIAGNOSIS AND DIFFERENTIAL DIAGNOSIS

An early presentation of Marfan's syndrome includes tall stature, ectopia lentis, scoliosis, mitral valve prolapse, aortic root dilation and aortic dissection. The diagnosis of Marfan's syndrome should be made in accordance with the revised diagnostic criteria, known as the Ghent nosology, which involves major and minor diagnostic findings (Chart 1)^26^ and is largely based on clinical manifestations from various organ systems and on the family history. A tall, thin body habitus, long limbs, arachnodactyly, pectus deformities and sometimes scoliosis with a positive family history in a young individual may be suggestive of a diagnosis of Marfan's syndrome.^27^

**Chart 4.** Major and minor diagnostic findings relating to Marfan's syndrome^26^

**Major criteria**
Enlarged aorta Tear in the aorta Dislocation of the lens Family history of the syndrome At least four skeletal problems, such as flat feet or curved spine Dural ectasia (enlargement of the lining that surrounds part of the spinal cord)
**Minor criteria**
Short sightedness (myopia) Unexplained stretch marks Loose joints

**Figure 1.**
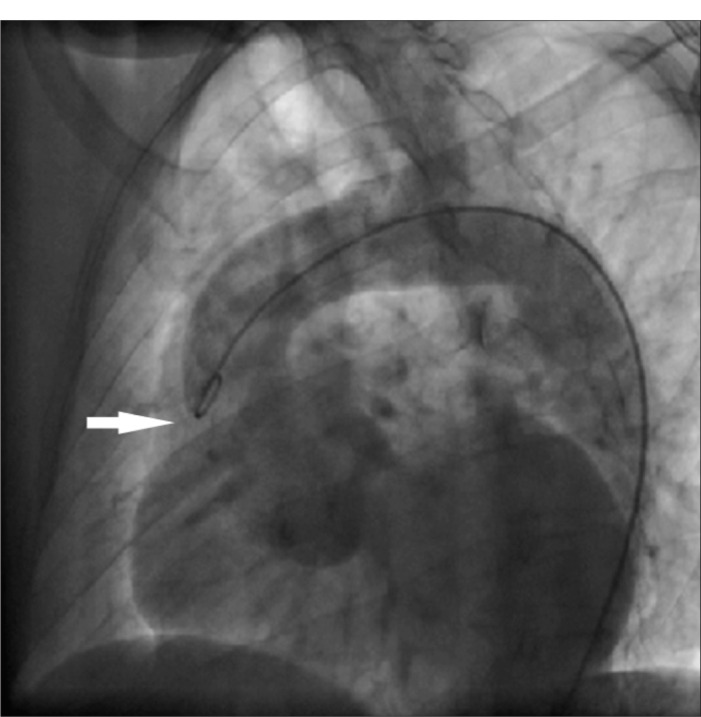
Aortogram showing dilated aortic root (arrow) of 5 cm in diameter, in a 44-year-old male patient with Marfan's syndrome.

Aortograms may show a dilated aortic root (Figure 1), or diffuse aneurysmal dilation of the ascending aorta in severe cases,^26^ There may be intimal flaps in the presence of aortic dissection.^28^ In the early years, aortography demonstrated aortic dilation and aortic regurgitation, but this may occasionally miss the diagnosis of aortic dissection.^29^

**Figure 2.**
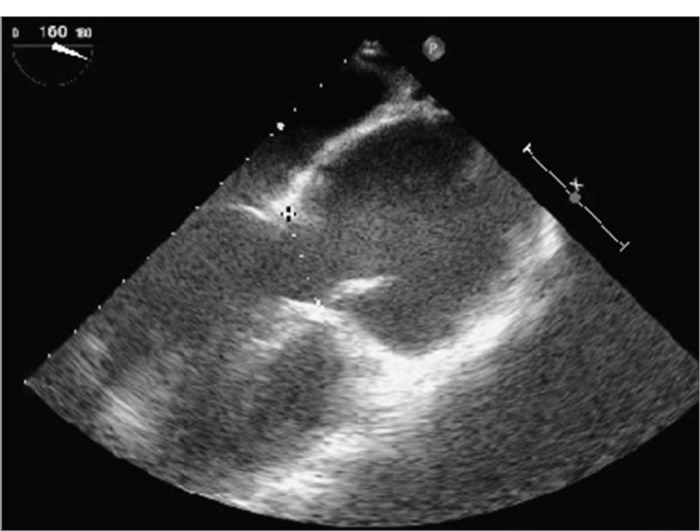
Echocardiography showing aortic root dilation to 67 mm in diameter on a long-axis view, in a 34-year-old male patient with Marfan's syndrome.

**Figure 3.**
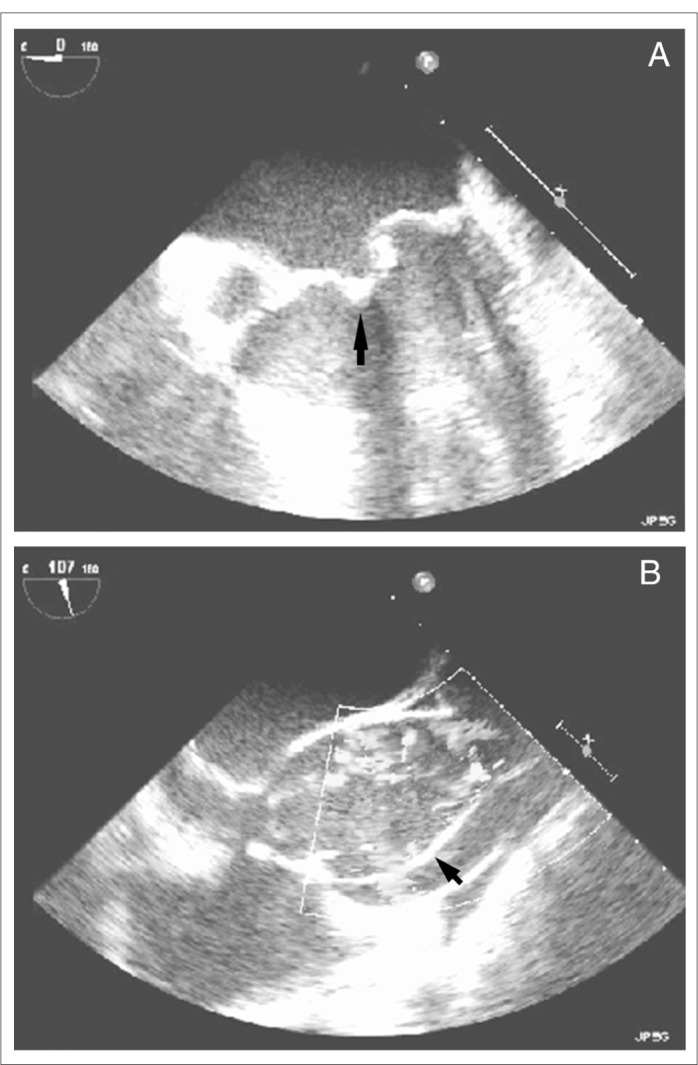
Echocardiography on a 29-year-old male patient with Marfan's syndrome showing (A) mitral valve prolapse (anterior mitral leaflet flail) (arrow) on a four-chamber view, and (B) aortic root dilation and aortic dissection with an intimal flap (arrow) in the aortic cavity on a long-axis view.

Echocardiography may detect aortic root dilation (Figure 2) and mitral valve prolapse (Figure 3A), the two main findings of Marfan's syndrome.^30^ In Marfan's patients, aortic root dilation may be present in 60%, mitral valve prolapse in 91% and aortic regurgitation in 23%. The incidence of aortic dilation and mitral prolapse in Marfan's syndrome has been found to be essentially equal in children and adults of the same sex.^13^ Transesophageal echocardiography and magnetic resonance imaging (MRI) are preferred over contrast aortography for diagnosing aortic dissection in pregnant patients with Marfan's syndrome.^31^ The use of radiation needs to be minimized, with adequate shielding for the fetus, if contrast aortography cannot be avoided.^31^ Ultrasound with higher sensitivity has demonstrated mild myocardial impairment in such patients.^32^ Color Doppler echocardiography is synergistically useful in diagnosing aortic dissection (Figure 3B) and it facilitates evaluation of the severity of aortic and mitral regurgitation that commonly complicates Marfan's syndrome. Color flow Doppler may assist in postoperatively diagnosing conduit leakage, coronary artery aneurysm, distal aortic dissections and prosthetic valve dysfunction.^33^ The risk of aortic dissection, which is the most serious manifestation of Marfan's syndrome, increases as the aorta enlarges. Therefore, elective composite graft surgery is recommended when the aortic root size reaches 60 mm, regardless of symptom status, or 55 mm in the presence of severe aortic regurgitation. Surgical replacement of the aortic root with a composite graft does not end the progress of the disorder.^33^

MRI delineates the presence and extent of aortic aneurysms and reveals the relationship between the aneurysm and arch vessels. It also demonstrates intimal flaps and individual lumina in types A and B aortic dissections.^34^ MRI has been found to be equivalent to computed tomography for depicting aortic, dural and hip abnormalities in patients who had not undergone surgery, but superior to computed tomography for postoperative evaluations on patients who received aortic valve replacement with Björk-Shiley or St. Jude valves, which preclude adequate evaluation of the aortic root on computed tomography scans.^35^ MRI may also facilitate measurement of the distances between the non-coronary, right coronary and left coronary cusps and the aortic root area on short-axis views of the aortic root. An asymmetrical aortic root might be of clinical importance in unexpected aortic root dissection.^36^

Morphological studies on the aponeurosis have revealed a collagen-type difference in Marfan, compared with the usual Dupuytren disease, with fetal type III in the former, in which the collagen bundles were more dispersed and less compact and resistant, and adult type I in the latter.^37^ Oxidative research has revealed that aortic 8-isoprostane was 32-50% greater in the Marfan group than in the control, and that SOD-1 and SOD-2 expressions were decreased in Marfan aortas, while xanthine oxidase, iNOS and the enzymatic subunits of NAD(P)H oxidase were increased.^38^ Genomic DNA analysis may occasionally miss the FBN1 mutations in Marfan patients.^39^ Reverse transcription PCR amplification of the fibrillin gene mRNA has detected a 123-bp deletion in affected individuals.^40^ In neonatal Marfan syndrome, FBN1 mutations have been noted in a region encompassing exons 24 to 32.^19^ Detection of enzymatic mutation is a more reliable and sensitive approach towards FBN1 mutation detection relating to Marfan's syndrome. In this way, causative mutations, such as R565X and R1523X, and polymorphisms that were missed by heteroduplex analysis have been observed.^41^ Work-ups through which a diagnosis of Marfan's syndrome can be made are listed in Table 2.

**Table 2.** Main diagnostic work-up for Marfan's syndrome

Work-up	Target
Echocardiogram, aortogram, magnetic resonance imaging and computed tomography	Measurement of the aortic root and detection of valve prolapse
Slit lamp examination	Lens abnormalities
X-ray studies on skeletal system	Evaluations of hand, spine, pelvis, chest, foot and skull for characteristic abnormalities
Magnetic resonance imaging	Dural ectasia
Prenatal testing	At approximately 10-12 weeks, using chorionic villus sampling, on a prospective parent who has Marfan syndrome
Genetic testing	Genetic testing may be helpful, but is very costly and time-consuming for different gene mutations

Among the differential diagnoses for Marfan's syndrome are homocystinuria, familial mitral valve prolapse syndrome, familial annuloaortic ectasia, isolated ectopia lentis, Ehlers-Danlos syndrome types II and III, Stickler syndrome (hereditary arthro-ophthalmopathy) and Klinefelter syndrome (Table 3).^42-50^

**Table 3.** Differential diagnoses of Marfan's syndrome^42-44^

Differential disorder	Expression	Sharing presentation	Differential presentation	Differential test	Etiology
Familial thoracic aortic aneurysm syndrome	Aortic root aneurysm and dissection	Aneurysms of the thoracic aorta	No eye or musculoskeletal findings. No systemic manifestations of Marfan's syndrome. Relevant family history in familial dissections.	No differentiating tests	Breakdown of the extracellular matrix proteins elastin and collagen by proteases such as collagenase, elastase, various matrix metalloproteinases, and plasmin (formed from plasminogen by urokinase plasminogen activator and tissue-type plasminogen activator)^45^
Bicuspid aortic valve and ascending aortic aneurysm	Maximum dilation often occurs further up in the ascending aorta, beyond the sinotubular junction	Occasionally occurs with Marfan's syndrome.	No eye or musculoskeletal findings	Echo, thorax computed tomography or MRI can reveal signs of bicuspid aortic valve.	Inadequate production of fibrillin-1
Ehlers-Danlos syndrome	An inherited heterogeneous group of connective tissue disorders, characterized by abnormal collagen synthesis, affecting skin, ligaments, joints, blood vessels and other organs. Marked joint hypermobility, papyraceous scars and mitral valve prolapse	Aortic aneurysm or aortic dissection at any age	Type IV variety most commonly affects the aorta, characterized by thin skin and bleeding disorders with increased bruising.	Skin biopsy for abnormal collagen and DNA testing for gene mutation.	EDS IV (mutations in the type III collagen gene), EDS VI (homozygous and compound heterozygous mutations in the lysyl hydroxylase gene), EDS VIIA and VIIB (mutations in the type I collagen genes), EDS VIIC (deficiency of procollagen N-proteinase) and EDS IX (decreased lysyl oxidase activity)^46^
Erdheim disease	Loss of elastic and muscle fibers in the aortic media, with accumulation of mucopolysaccharide, sometimes in cyst-like spaces between the fibers.^47^	Aortic root dilation or rupture	No eye or musculoskeletal findings or family history.	No differentiating tests	
Homocystinuria	Marfanoid habitus, ectopia lentis, mental retardation and osteoporosis	Ectopia lentis	A deficiency of cystathionine β synthase. Tall stature, long-bone overgrowth and ectopia lentis, but typically without aortic enlargement or dissection.	Raised concentrations of plasma homocystine	Mutations in the CBS, MTHFR, MTR and MTRR genes^48^
Loeys-Dietz syndrome	Loeys-Dietz syndrome is a genetic disorder that affects blood vessels in the body, especially the aorta. It was first described in the medical literature in January 2005.	Aortic dilation, aneurysm and dissection, and mitral valve prolapse	No associated lens dislocation. Aortic dissection occurs at much smaller diameter. Bifid uvula or cleft palate, arterial tortuosity and hypertelorism	Not currently available	Mutations in the genes encoding transforming growth factor beta receptor 1 (TGFBR1) or 2 (TGFBR2)
Shprintzen-Goldberg syndrome	Craniosynostosis (involving the coronal, sagittal or lambdoid suture), distinctive craniofacial features, skeletal abnormalities (dolichostenomelia, arachnodactyly, camptodactyly, pes planus, pectus excavatum, pectus carinatum, scoliosis, joint hypermobility or contractures), neurological abnormalities, mild-to-moderate mental retardation and brain anomalies	Mitral valve prolapse, mitral regurgitation and aortic regurgitation	Aortic root dilation is most likely not found.	Not available	Uncertain, but maybe fibrillin-1 mutations.
MASS phenotype	Mitral valve prolapse, mild aortic dilation, striae atrophicae and skeletal involvement	Mitral valve prolapse, aortic root dilation and skin and skeletal conditions	Long limbs, deformity of the thoracic cage, striae atrophicae, mitral valve prolapse and mild and non-progressive dilation of the aortic root	Careful follow-up is needed to distinguish the MASS phenotype from emerging Marfan's syndrome, especially in children.	Fibrillin-1 mutations
Congenital contractural arachnodactyly (Beals syndrome)^49^	Multiple flexion contractures, arachnodactyly, severe kyphoscoliosis, abnormal pinnae and muscular hypoplasia	Skeletal features with Marfan's syndrome such as marfanoid habitus, arachnodactyly, camptodactyly and kyphoscoliosis	Multiple joint contractures (especially elbow, knee and finger joints), and crumpled ears in the absence of significant aortic root dilation are characteristic of Beals syndrome and rarely found in Marfan syndrome.	Crumpled appearance of ear helix and congenital contractures, typically without ocular and cardiovascular complications	A mutation in the fibrillin-2 gene on chromosome 5q23
Isolated ectopia lentis	A rare syndrome characterized by dislocation of eye lenses, which often occurs at birth.	Ectopia lentis, maybe associated with mild skeletal findings.	Mental retardation, impaired joint mobility and stiff joints, but without cardiovascular abnormalities	No	Fibrillin-1 mutations
Stickler syndrome (hereditary arthro-ophthalmopathy)	A relatively common genetic disorder characterized by very flexible (hyperextensible) joints, typical facial characteristics, hearing loss and severe nearsightedness with associated eye problems.	Tall stature, mitral valve prolapse and retinal detachment	Retrognathia, midfacial hypoplasia, without aortic involvement	No	Mutation in the COL2A1 gene on chromosome 12 in region 12q13.11-q13.2
Klinefelter syndrome (47, XXY or XXY syndrome)	Men with Klinefelter syndrome present with sequelae of hormonal and spermatogenic testicular failure, such as infertility, low testosterone, erectile dysfunction and low bone mineral density	Marfanoid habitus	Small testes and genitalia, and learning difficulty	Serum FSH levels were elevated ^50^	FGFR3 gene

MRI = magnetic resonance imaging

## PREGNANCY

Pregnant women with Marfan's syndrome have the potential to present acute aortic dissection, especially under conditions of aortic root dilation^51^ and fetal death,^20^ or in cases of inheritance of this disease by the child.^52^ Complications such as rapid aortic dilation and aortic dissection may occur at any time during pregnancy or even postpartum, but most often in the third trimester.^53^ Cesarean section is preferred in women with aortic dilation.^26^ An aortic root diameter of less than 40 mm would result in favorable maternal and fetal outcomes.^54^ The fetus has a 50% chance of acquiring the disease.^55^ Cardiopulmonary bypass during pregnancy is associated with maternal mortality of 3% and fetal mortality of 20%.^56^

## MANAGEMENT

It was recently suggested that the current strategies for Marfan's management should be blood pressure control and restrictions on physical activities.^57^ Nevertheless, Marfan's syndrome can be effectively managed if it is under integrated care provided by a team of specialists from all relevant specialties.^58^

Improvements in life expectancy could be achieved more readily through management of cardiovascular disorders, including mitral valve prolapse, aortic dilation and aortic dissection. The treatment may include prophylactic β-blockers to slow down the dilation of the ascending aorta, and prophylactic aortic surgery. Short to midterm follow-up results have suggested that β-blockers are useful for preventing progressive dilation of the aorta.^59^ The mean slope of the regression line for the aortic root dimensions has been found to be significantly lower in the treatment group than in the controls.^59,60^ Recent research on the etiology of Marfan's syndrome in mouse models has suggested that aortic root dilation and fibrillin-1 abnormalities are probably caused by excessive TGF-β signaling, and that TGF-β antagonists, including angiotensin II-receptor blockers, may significantly slow down the progression of aortic root dilation^61^ or prevent certain manifestations of Marfan's syndrome, including aortic aneurysm.^44^ Ahimastos et al.^62^ observed that perindopril therapy may reduce arterial stiffness, central and peripheral pulse wave velocities, aortic root diameters (in both end-systole and end-diastole) and TGF-β, among patients with Marfan's syndrome in comparison with placebo.

Prophylactic aortic root replacement with a composite graft is recommended when aortic root dilation predisposes towards aortic rupture and the potential aortic dissection could theoretically have been prevented, as suggested by current guidelines: (1) aortic root diameter ≥ 55 mm; (2) positive family history of aortic dissections and aortic root diameter ≥ 50 mm; and (3) aortic root growth ≥ 2 mm/year.^11^

Aortoplasty with aortic valve replacement has been successful in infants with Marfan's syndrome who presented annuloaortic ectasia and aortic regurgitation.^63^ However, the Bentall operation is a preferred procedure for Marfan patients. From this, low operative mortality and long-term survival can be expected, with a survival rate of 80% after five years and 60% after 10 years. Clinical observations have shown that composite valve replacement and valve-sparing repair procedures were associated with early postoperative mortality rates of 6.8% and 0%, respectively.^64^ Gillinov et al.^65^ reported, among patients with Marfan's syndrome undergoing aortic root replacement, that the mean aortic root diameter was 6.2 ± 0.2 cm. Aortic root replacement was carried out in 96% of the cases, and a mitral valve procedure in 42%, with a 10-year survival rate of 79% ± 10%. Alexiou et al.^66^ found that the operative mortality rate was 6.1%, while the procedure led to 10-year freedom from thromboembolism, hemorrhage and endocarditis of 88%, 89.8% and 98.4%, respectively. de Oliveira et al.^67^ achieved satisfactory freedom from reoperation, which was 75% after 10 years for root replacement and 100% for the valve-sparing patients, and excellent 10-year survival, which was 87% and 96% for the two procedures, respectively.

## CONCLUSIONS

Marfan's syndrome is a rare hereditary connective tissue disorder affecting many parts of the body. Establishment of a diagnosis of Marfan's syndrome is based on the Ghent nosology, which involves comprehensive evaluation of major and minor systemic manifestations. The pathogenesis of Marfan's syndrome has not been fully elucidated, but fibrillin-1 gene mutations are believed to exert a dominant negative effect through excessive TGF-β signaling pathways. Cardiovascular malformations, chiefly aortic root dilation and mitral valve prolapse, are the most life-threatening symptom of Marfan's syndrome, since these patients are at risk of acute aortic dissection. Prophylactic aortic root replacement with a composite graft is recommended when the dilated aortic root has a tendency to rupture and the potential aortic dissection could theoretically have been prevented. Regular cardiovascular, ocular and skeletal surveillance by means of echocardiography, slit lamp examination of the eye, and magnetic resonance is recommended upon diagnosis or after the operation.
